# A Case of Empyema Due to Actinomyces odontolyticus and Streptococcus Species Co-infection in an Immunocompetent Woman

**DOI:** 10.7759/cureus.32700

**Published:** 2022-12-19

**Authors:** Diego P Peralta, Haya Najjar

**Affiliations:** 1 Division of Infectious Diseases, Texas Tech University Health Sciences Center El Paso, El Paso, USA; 2 Department of Internal Medicine, University of California San Diego, San Diego, USA

**Keywords:** co-infection, immunocompetent, empyema, actinomyces odontolyticus, streptococcus species

## Abstract

Actinomyces species are opportunistic pathogens, difficult to isolate, and often accompanied by other pathogens. We report the case of an immunocompetent woman who presented with respiratory distress and was discovered to have a right-sided empyema requiring chest tube drainage. *Streptococcus *species and *Actinomyces odontolyticus* were isolated in pleural fluid cultures. Initial empiric broad-spectrum antibiotic therapy and chest tube placement failed to show clinical improvement. Upon isolation of Actinomyces, the treatment was streamlined to ampicillin/sulbactam while pleural drainage continued, producing significant clinical status improvement in the patient. Given the known co-pathogenicity of Actinomyces species and the difficulty in isolating Actinomyces, it is essential to consider antibiotic coverage for Actinomyces species in those with Streptococcus species empyema.

## Introduction

Actinomyces species are filamentous, slow-growing, anaerobic Gram-positive rods that are part of the oral, gastrointestinal, and vaginal floras. The disruption of tissue barriers promotes pathogenicity, and infection is known to transgress tissue planes. Presentations of actinomycosis are usually indolent, chronic, and refractory or relapsing, as complete eradication requires prolonged antibiotic therapy [1]. There are challenges in diagnosis, primarily due to difficulty in isolation. When isolated, it is usually accompanied by other pathogens, one of the most common being Streptococcus species [2,3]. Depending on the clinical response, aminopenicillin agents are used as the primary treatment for actinomycosis for a prolonged duration [4,5]. We report a case of empyema caused by a co-infection of *Actinomyces odontolyticus* and *Streptococcus* species in an otherwise healthy young woman.

## Case presentation

A 30-year-old woman was transferred to our institution from Mexico with a three-week history of midsternal chest pain radiating to the right upper back associated with fever, shortness of breath, and decreased appetite. Her medical history was unremarkable and negative for recurrent childhood infections or previous hospitalizations. She had no exposure to sick people, animals, chemicals, or hazards. Alcohol, drug, and tobacco use screening was negative, but she reported secondhand smoking exposure. Before transference to our hospital, she was hospitalized for a week in a hospital in Mexico, where she was found to have right-sided empyema. Her management included the placement of a chest tube and seven days of empiric intravenous vancomycin and levofloxacin. However, she did not show clinical improvement with those interventions. Therefore, the patient requested to be transferred to the U.S. for further medical care. A pleural fluid sample collected during chest tube placement was sent for culture, but results were unavailable at the time of her transfer.

On admission to our hospital, the examination revealed an obese woman (BMI 38) with a temperature of 37.9 °C, a heart rate of 112 beats per minute, a respiratory rate of 19 breaths per minute, and a blood pressure of 118/55 mmHg. She was in respiratory distress, with labored breathing requiring 5 L of oxygen by nasal cannula. The oral exam showed no cavities or lesions. In the pulmonary exam, there were decreased breath sounds and dullness to percussion over the right lung field, whereas the left lung field was clear to auscultation. The previously placed chest tube was draining purulent fluid.

**Figure 1:**
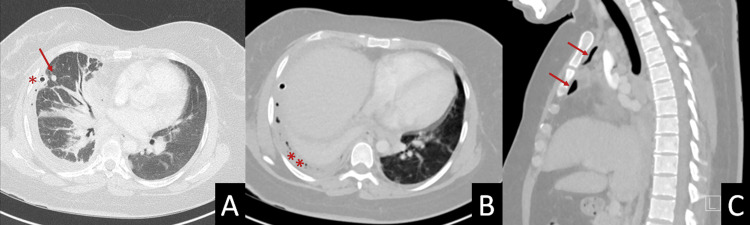
CT thorax (A) thoracostomy tube (asterisk), pulmonary nodule (arrow), (B) pleural effusion with air bubbles (asterisks), and (C) pneumomediastinum (arrows).

Initial blood work showed elevated ferritin, a normal leukocyte count, high creatinine, and elevated liver enzymes (Table 1). The CT thorax showed a pleural effusion, hydropneumothorax, atelectasis, nonspecific nodules, and interstitial edema on the right side. There were left-sided nonspecific airspace opacities and minimal pneumomediastinum with mildly enlarged lymph nodes (Figure 1). At our facility, a pleural fluid sample was collected for analysis and culture. The analysis was consistent with an exudate (Table 2).

**Table 1:** Blood work results *Interpretation: ≤0.5 ng/mL: systemic infection not likely; ≤2 ng/mL: systemic infection possible; >2 ng/mL: systemic infection likely ≥10 ng/mL: systemic infection very likely.

Test	Reference range	Day 1	Day 4	Day 6	Day 10
White blood cells	4.5–11.0 × 10^3^/µL	7.51	15.88	11.39	7.98
Creatinine	0.52–1.04 mg/dL	0.50	0.70	0.90	1.0
AST	14–36 U/L	28		18	27
ALT	0–35 U/L	21		14	26
Ferritin	6.24–137 ng/mL	372			
Lactic acid	0.7–2.1 mmol/L		0.6		
Procalcitonin			26.62*		

**Table 2:** Pleural fluid analysis LDH: lactate dehydrogenase; TP: total protein; ADA: adenosine deaminase (*normal value <9.2 U/L).

	Units	Pleural fluid analysis #1	Pleural fluid analysis #2
Color		Yellow	Orange
Appearance		Turbid	Turbid
White blood cells	#/µL	431,500	400,920
Red blood cells	#/µL	44,000	340
Neutrophils	%	98%	98%
Glucose pleural fluid	mg/dL	<20	<20
Glucose serum	mg/dL	102	97
LDH pleural fluid	U/L	>8600	>8600
LDH serum	U/L	166	216
TP pleural fluid	g/dL	4.2	5.0
TP serum	g/dL	6.0	6.6
pH		6.5	N/A
ADA	U/L*	570.2	496.3

**Figure 2:**
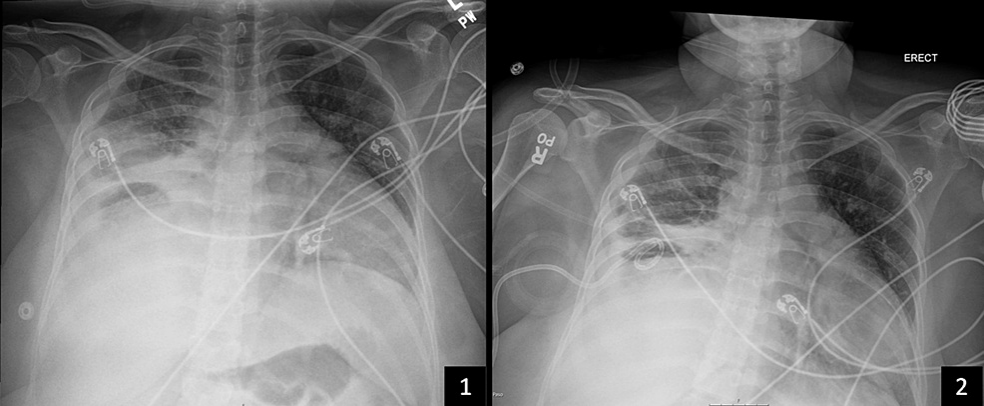
Chest X-ray images showing placement of pigtail catheter (1) Before placement and (2) after placement.

The patient was started empirically on intravenous piperacillin/tazobactam and vancomycin and is awaiting culture results and medical records from the hospital in Mexico for review. On the second day of hospitalization, she accidentally removed her chest tube. In the following 48 hours, she developed persistent fever, tachycardia, and worsening tachypnea with increasing oxygen requirements, prompting her transference to the medical ICU for close monitoring. Her leukocyte count also increased, and her procalcitonin level was significantly elevated (Table 1). Given her clinical deterioration and lack of source control, as noted in chest X-rays by a more extensive right pleural effusion, a pigtail catheter was placed on the fourth day (Figure 2). A new pleural fluid sample was obtained for analysis and culture during catheter placement. Analysis showed a persistent exudative pattern (Table 2). Similar to the first pleural fluid sample, there was an elevated adenosine deaminase (ADA) level; however, pleural fluid acid-fast stains and mycobacterial cultures were negative. She spent 48 hours in the ICU, temporarily requiring a non-rebreather mask before transitioning back to a nasal cannula.

**Figure 3:**
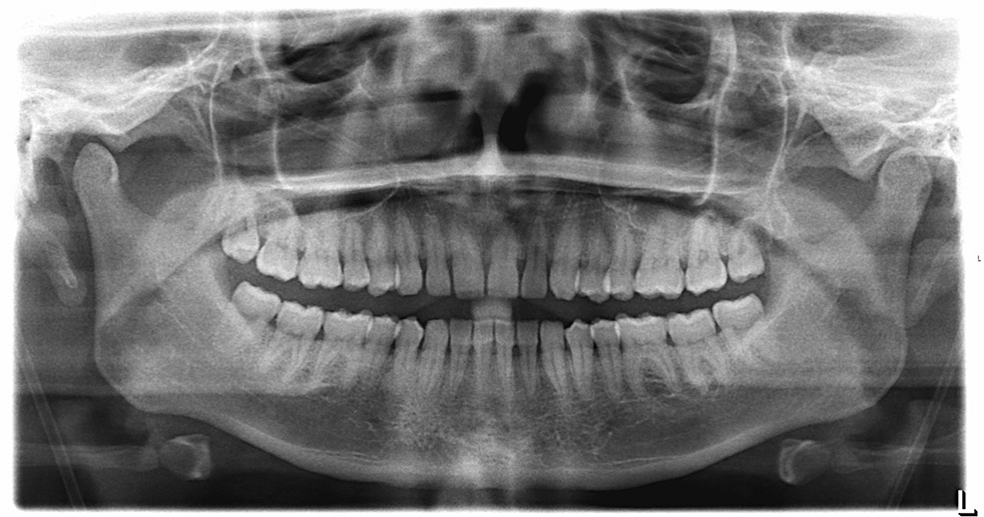
Panorex X-rays No abnormalities of the mandible, paranasal sinuses, and teeth are seen.

On the fifth day, she was transferred back to the medical ward, where she contacted the physician who took care of her in Mexico and notified her that the pleural fluid culture yielded Streptococcus species. An official culture report was eventually obtained and reviewed, showing a pan-susceptible Streptococcus species isolate that was not further identified (Table 3). The same day, the first pleural fluid anaerobic culture was reported positive for Actinomyces species growth and later identified as Actinomyces odontolyticus by matrix-assisted laser desorption/ionization-time of flight (MALDI-TOF) mass spectrometry (MS). Antimicrobial susceptibilities were not performed because our hospital does not have the capability to perform anaerobe susceptibilities. Malignancy was ruled out by the cytologic evaluation of the pleural fluid. The blood and second pleural fluid sample cultures were negative. The HIV screening test was also negative. Panorex X-rays obtained to assess for a possible oral source of infection were unrevealing (Figure 3).

**Table 3:** Streptococcus species susceptibilities Susceptibility report obtained from the hospital in Mexico. *MIC: minimum inhibitory concentration.

Antibiotic	MIC* (µg/mL)	Interpretation
Clindamycin	<2	Susceptible
Ciprofloxacin	<2	Susceptible
Norfloxacin	<2	Susceptible
Levofloxacin	<1	Susceptible
Vancomycin	<0.5	Susceptible

**Figure 4:**
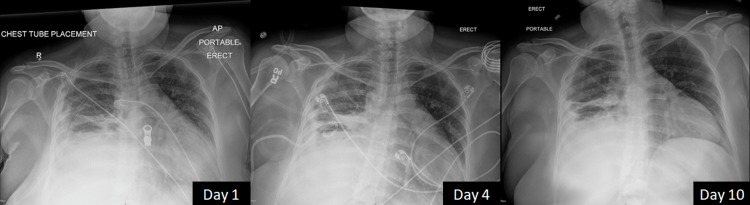
Serial chest X-rays Hospital day 1: chest tube placed in Mexico can be seen. Hospital day 4: image after the new pigtail catheter placement. Hospital day 10: pigtail drain removed and stabilization of empyema can be seen.

Upon identification of Actinomyces odontolyticus and known susceptibilities of Streptococcus species isolated in Mexico, the antimicrobial therapy was transitioned to ampicillin/sulbactam. In the subsequent days, the patient showed significant clinical improvement. Fever, tachypnea, and leukocytosis resolved with down-trending oxygen requirements by day eight. Serial chest X-rays showed stabilization in the size of the empyema (Figure 4). The patient was on room air by day 10, and after minimal drainage output, the pigtail catheter was removed. She was discharged with oral amoxicillin/clavulanate for an anticipated duration of six to twelve months, depending on her clinical response and resolution of pulmonary abnormalities. Outpatient follow-up was arranged; however, she never showed up at the clinic.

## Discussion

Isolation of Actinomyces odontolyticus from the pleural fluid is uncommon, with a limited number of cases reported worldwide [6,7]. Among these cases, tobacco and alcohol use, poor oral hygiene, and underlying pulmonary disease were the most common risk factors. The most common sources of infection found were dental disease and aspiration [7], and they were more often seen in middle-aged men [7,8]. Our patient was a young woman without any chronic medical conditions or the risk factors mentioned above. Although Actinomyces odontolyticus was isolated in cultures, an evident source of infection was never identified. We believe that the incidence of empyema from Actinomyces species is underestimated, given the difficulty of its isolation. Antibiotic use, inhibition by commensal flora, inadequate culture conditions, and prolonged incubation periods make isolation challenging [9,10].

The isolation of Actinomyces odontolyticus greatly impacted the clinical course of our patient as it redirected her antibiotic therapy from a broad-spectrum regimen to a streamlined treatment. In the setting of source control, after the initial placement of a chest tube for drainage, treatment with levofloxacin, vancomycin, and piperacillin/tazobactam failed to show appropriate clinical improvement. Her condition worsened, requiring more oxygen supplementation and a brief stay in the medical ICU. Once the pathogen was identified, a new pigtail was placed, the antibiotic therapy was changed to ampicillin/sulbactam, and her clinical condition improved rapidly.

Another etiology to consider was tuberculosis, given the elevated levels of ADA in pleural fluid samples in a patient originally from Mexico. However, the mycobacterial workup was negative, and her positive clinical response to antibiotic therapy and source control was reassuring. Elevated levels of ADA can also be seen in typhoid fever, infectious mononucleosis, liver disease, sarcoidosis, leukemia, brucellosis, acute pneumonia, parapneumonic effusions or empyemas, rheumatoid arthritis, and malignancies. Therefore, ADA is not the most suitable marker for differentiating between pulmonary tuberculosis and other pulmonary infections [11].

Actinomyces species are susceptible to penicillins, carbapenems, macrolides, and lincosamides and are resistant to quinolones and metronidazole [4]. Aminopenicillins such as ampicillin and amoxicillin are the preferred first-line regimen as they are almost uniformly active against Actinomyces species [4,5]. Although piperacillin/tazobactam and ceftriaxone can be used against Actinomyces species, they are usually not the first-line regimen due to their broad spectrum, and some isolates have shown high-level resistance to them [5]. We suspect the Actinomyces odontolyticus isolate was resistant to piperacillin/tazobactam, given the change in the clinical course of our patient when ampicillin/sulbactam was used. The suspicion could not be proven due to our hospital's lack of anaerobic culture testing capabilities. A prolonged duration of therapy, between six and twelve months, is recommended in actinomycosis due to the high frequency of recurrence [2,3,12]. Some reports demonstrate successful treatment with a shorter duration of therapy; however, more data are needed to confirm efficacy [12,13].

Actinomyces species are often part of polymicrobial infections. Streptococcus species are one of the most common co-pathogens [2,3]. At our hospital, Streptococcus was not isolated in the pleural fluid cultures obtained, likely due to its susceptibility to the antibiotics our patient received before transference. We strongly suspect a co-infection by both pathogens, given the lack of improvement at the other hospital despite the therapy implemented. Two studies showed that co-aggregates of Actinomyces viscosus and Streptococcus species have greater pathogenicity and incidence of abscess formation than either pathogen alone [14,15]. The co-aggregates were also more resistant to phagocytosis by neutrophils in vitro and in vivo, suggesting a potential synergistic mechanism [14,15]. This observation suggests an underlying process for the pathogenicity of Actinomyces species in the presence of Streptococcus species. Isolation of Streptococcus species in empyema refractory to treatment may hint that another pathogen, such as Actinomyces, is present.

Most cases of actinomycosis are not diagnosed until an advanced phase, likely due to difficulty in isolation, low suspicion of the pathogen, and, often, misleading suspicion of malignancy given its ability to invade tissues [2,3,8,9]. Delay in diagnosis and treatment increases the risk of complications, such as empyema necessitans or extensive loculations requiring management with cardiothoracic surgery [7,10]. Our patient achieved clinical improvement with appropriate antibiotic therapy and source control through the placement of chest drains, avoiding major surgical interventions.

## Conclusions

The diagnosis of Actinomyces infections is challenged by the difficulty of its isolation and being often part of polymicrobial infections. It is, therefore, essential to consider Actinomyces co-infection in a patient with a streptococcal empyema refractory to initial treatment. The selection of an antibiotic regimen that provides coverage for Actinomyces species in such cases may ultimately impact clinical outcomes. Although there are several antimicrobial options to treat actinomycosis, aminopenicillin agents remain the preferred first-line regimen. Reports of emerging resistance to second-line agents have been described, underpinning the value of anaerobe susceptibility testing.
